# Study on the Spatiotemporal Evolution and Influencing Factors of Urban Resilience in the Yellow River Basin

**DOI:** 10.3390/ijerph181910231

**Published:** 2021-09-28

**Authors:** Yu Chen, Xuyang Su, Qian Zhou

**Affiliations:** 1 School of Economics and Management, Zhengzhou University of Light Industry, Zhengzhou 450000, China; 2012030@zzuli.edu.cn (Y.C.); suxuyang_123@163.com (X.S.); 2 Economics School, Zhongnan University of Economics and Law, Wuhan 430073, China

**Keywords:** urban resilience, spatiotemporal differentiation, ESDA, geographical detector model, YRB

## Abstract

The outbreak of COVID-19 has prompted consideration of the importance of urban resilience. Based on a multidimensional perspective, the authors of this paper established a comprehensive evaluation indicator system for evaluating urban resilience in the Yellow River basin (YRB), and various methods such as the entropy value method, Theil index, exploratory spatial data analysis (ESDA) model, and geographical detector model were used to measure the spatiotemporal characteristics and influencing factors of urban resilience in the YRB from 2011 to 2018. The results are as follows. (1) From 2011 to 2018, the urban resilience index (URI) of the YRB showed a “V”-shaped dynamic evolution in the time series, and the URI increased by 13.4% overall. The resilience of each subsystem showed the following hierarchical structure: economic resilience > social resilience > ecological resilience > infrastructure resilience. (2) The URI of the three major regions—upstream, midstream, and downstream—increased, and the resilience of each subsystem in the region showed obvious regional characteristics. The comprehensive difference in URI values within the basin was found to be shrinking, and intraregional differences have contributed most to the comprehensive difference. (3) There were obvious zonal differences in the URI from 2011 to 2018. Shandong Peninsula and Hohhot–Baotou–Ordos showed a “High–High” agglomeration, while the southern and southwestern regions showed a “Low–Low” agglomeration. (4) Among the humanist and social factors, economic, fiscal, market, urbanization, openness, and innovation were found to be the factors that exert a high impact on the URI, while the impacts of natural factors were found to be low. The impact of the interaction of each factor is greater than that of a single factor.

## 1. Introduction

The establishment of an urban system is randomly affected by changes in the internal and external environment as a consequence of the nonlinear interaction of multiple factors such as economic and social change, cultural adaptation, and resource integration [1], which forms a complex and interconnected geographic entity [2]. At present, COVID-19 has caused immeasurable economic losses, social impacts, and casualties worldwide. This is not only a major public health crisis but also a test of urban disaster risk emergency management. From the perspective of temporal and spatial scales, factors such as population, capital, information, energy, and resources continuously flow among cities and promote the rapid development of urbanization. The essence of urbanization is population migration [3]; in 2020, 56.2% of the world’s population lived in urban areas, and it is expected that this proportion will increase to 62.5% by 2035 [4]. Exponential population growth, sustained economic development, and excessive resource consumption place tremendous pressure on urban systems, making cities extremely vulnerable to human–natural issues such as negative externalities, climate change, and various disasters [2,5]. Uncertainty and unknown risks have gradually become bottlenecks that restrict urban survival and sustainable development and directly or indirectly threaten the safety and quality of life of urban residents. Maintaining the initial core structure and basic functions under various unpredictable disturbances and pressures [1,6], improving the adaptability of the urban system, enhancing resistance to shocks and the ability to recover from disasters, and minimizing the adverse effects of perturbation factors have become urgent issues in the process of urbanization [7]. China’s “14th Five-Year Plan” proposed building liveable, innovative, smart, green, humane, and resilient cities; enhancing the capacity of public facilities to cope with storms, droughts, and geological disasters; and improving emergency shelter functions in public facilities and buildings. Resilient city construction has attracted wide attention from academics, social organizations, and government departments, as well as becoming a crucial topic in the field of urban geography and planning.

As a new model and concept of urban disaster prevention and mitigation, many scholars have discussed urban resilience from qualitative and quantitative point of views, and the relevant research has mainly focuses on the following:

(1) Concept definition: The term resilience originated from the Latin “resilio” [8]. In the 1990s, the theory of resilience was creatively introduced into urban planning and construction, expanding the horizons of urban disaster research [7]. Urban resilience is usually recognized as the preparation and planning by urban systems for unfavourable factors, with absorption, recovery, and better adaptability in the face of disturbances [9] and complex and dynamic characteristics in the development process [10]. To enhance the short-term response capacity and long-term adaptability of urban systems [11], resilient cities can mitigate and prevent disaster risks; minimize loss or disadvantage to life, property, infrastructure, economic activities, and the environment from potential threats [12]; effectively guarantee the integrity and liveability of urban systems; and ensure the effective operation of functions in changing socioeconomic and environmental conditions [13]. Although there is no unified and recognized notion of urban resilience in academia, the construction of resilient cities is considered to be a new way to guide the development of sustainable cities [5], which should have three important abilities: the ability to absorb various pressures and maintain a stable state, the ability to self-organize, and the ability to adapt and learn [14]. Urban resilience is also characterized by biodiversity, versatility, multiscale networks, modularity, and adaptive design [15].

(2) Evaluation system and method selection: Building a reasonable evaluation index system based on the multidimensional perspective is the basis of quantitative research on urban resilience. Many scholars have chosen indicators from several dimensions of economy, society, infrastructure, institutions, and natural environment [16,17,18], using models such as the system dynamics [19,20], performance credit card [10], resilience maturity model [21,22], situation analysis [23], and “Scale–Density–Morphology“ evaluation models [14] to measure the level of urban resilience or explore a certain dimension of it, such as economic resilience [24], social resilience [25], ecological resilience [26], or infrastructure resilience [27]. In addition, some scholars have used the propensity score matching and difference in difference model to explore the role of smart city construction in improving resilience [28].

(3) Influencing factors: The improvement of the urban resilience level is subject to the combined effect of multiple factors, and identifying the leading factors behind such resilience is conducive to guiding the planning and construction of resilient cities. From both internal and external perspectives, the factors affecting urban resilience are mainly explored from the human and natural perspectives, and theoretical frameworks of urban resilience such as PEOPLES [22], DROP [29], RCPF [30], and HES [6] have been established through various methods to explore the influence of social [31], economic [32], regime [29], and ecological environment [33] on urban resilience. These empirical studies have systematically analysed the reasons for differences in urban resilience at different scales across the country, urban agglomerations, or provinces, and they have proposed specific resilient city construction paths for different regions.

In summary, scholars have made fruitful achievements in the study of urban resilience, but there remain problems that need to be explored in depth. First, current studies are mostly focused on qualitative research such as concept introduction and theoretical exploration, with less extensive quantitative research focused on certain dimensions of urban resilience, such as urban economic, social, ecological, or infrastructure resilience. Research on comprehensive urban resilience is neither thorough nor systematic. Second, the influencing factors of urban resilience are mainly selected from humanistic and economic factors, and the impact of natural factors on urban resilience has not been fully considered. As a giant system with a complex enmeshment of “economy–society–nature”, it is more reasonable to judge the influencing factors from the perspective of humanity and nature. Finally, existing studies are mostly concentrated at the national, provincial, and urban agglomeration levels, while research on watershed areas, which are regions with significant spatial heterogeneity, is relatively insufficient.

The YRB is the birthplace of Chinese civilization. In 2019, the ecological protection and high-quality development of the YRB was determined as a national strategy, and the region has a very important position in the construction of a new domestic and international economic dual-cycle development pattern. Compared with the Yangtze River Basin, the YRB spans multiple natural subregions and is a typical area with rapid changes in economics, society, and environment [34]; multiple fragile ecosystems in the basin have produced a relatively close spatial coupling. The uneven distribution of water and land resources and the shortage of water resources comprise the main contradiction that leads to the tense relationship between man and land, as well as causing the poor navigation conditions of the Yellow River. Most of the regional economic development is organized through a “centre–periphery” structure, and the main trend of industrial population flow and distribution is formed by relying on the main traffic axis. There are obvious differences in urban economic development strength, natural resource endowments, and traffic location conditions in the basin. The construction of urban resilience in this area is relatively insufficient, and its resilience is inadequate in the face of various disasters and risk intrusions. For example, Zhengzhou in the lower reaches of the Yellow River is a national central city and an important transportation hub. The extreme weather on 20 July 2021 caused serious waterlogging, traffic paralysis, and casualties in the city. Water and power outages in some areas caused inconvenience to residents’ daily lives. The socioeconomic and ecological system is somewhat fragile, which severely restricts regional coordination and linkage and sustainable development. Therefore, it is necessary to measure the urban resilience level in the basin and clarify its driving factors. Based on this, the authors of this paper used the entropy method to measure the urban resilience index and then analysed the spatial heterogeneity and imbalance of urban resilience development by using spatial autocorrelation and the Theil index. Finally, the geographical detector model was used to explore the influencing factors of urban resilience. The results of this study are expected to provide reference for urban planners and decision makers in the construction of resilient cities.

## 2. Methods and Data Description

### 2.1. Construction of Evaluation System

Urban resilience is the extent to which an urban system can withstand and absorb the impact of various uncertain factors, as well as the ability to adapt, recover, and learn when dealing with disturbances. Based on the relevant literature, 24 specific indicators from four dimensions (economic resilience, society resilience, ecological resilience, and infrastructure resilience) were established to measure the URI of the YRB. The index system is attached in Appendix A (Table A1).

Economic resilience is embodied in the adaptability and stability of urban economic systems when facing the impact of unknown risks [24]. A highly resilient urban system requires a similarly strong level of economic development and has the ability to quickly overcome the crisis and resume production in response to external disturbances. Accordingly, six indicators were selected from the aspects of economic aggregate, financial security, economic structure, financial capital, investment intensity, and economic growth to reflect a city’s comprehensive economic strength, economic diversity, and stability. 

Social resilience reflects a city’s health security and emergency management when it suffers short or long-term disturbance, with a focus on creating a high-quality social environment with development potential [25]. We selected six indicators from the aspects of education level, medical investment, residence income, employment structure, unemployment structure, and health protection to reflect a city’s human capital and the ability to resolve risks. 

Ecological resilience is reflected in the resilience of an urban ecological environment when facing excessive emissions of pollutants and reduction of green space that lead to environmental overload. It is an important factor of urban resilience [35]. For this, we selected six indicators from the aspects of environmental conservation level, urban greening level, waste utilization, environmental remediation, environmental pollution pressure, and waste emission intensity that reflect the service capacity, governance capacity, and pressure of an ecological environment. 

Infrastructure resilience reflects the ability of a city to protect people, evacuate, and communicate with others during disasters or risks. It is at the forefront of crises response [36]. Six indicators from the aspects of infrastructure construction, level of transportation facilities, engineering support capability, internet penetration, electricity development level, and communication sophistication were selected to reflect infrastructure resilience.

### 2.2. Research Methods

#### 2.2.1. Entropy Value Method

The entropy value method is an objective comprehensive evaluation method that can effectively avoid human interference [37] and that many scholars have applied to comprehensive evaluation. To better reflect the role of negative indicators, the extreme value standardization method was used to nondimensionally process the data of various indicators, and the URI was calculated according to the linear fitting formula. The specific calculation steps are as follows [35].

First, according to the positive and negative indicators of urban resilience, the range method was used to standardize the original data:

(1) $$\mathrm{Positive}\;\mathrm{indicator}:\;\;x_{ij}^{\prime }=\frac{x_{ij}-\min(x_{ij})}{\max(x_{ij})-\min(x_{ij)}}$$

(2) $$\mathrm{Negative}\;\mathrm{indicator}:\;\;x_{ij}^{\prime }=\frac{\max(x_{ij})-x_{ij}}{\max(x_{ij})-\min(x_{ij})}$$

where $x_{ij}$ is the original value of the *i*-th evaluation object corresponding to the *j*-th index; $x_{ij}^{\prime }$ is the standardized value; $\max(x_{ij})$ and $\min(x_{ij})$ are the maximum and minimum values of each index, respectively.

Second, the entropy value of the *j*-th index was calculated:

(3) $$e_{j}=-k\overset{n}{\underset{i=1}{\sum }}P_{ij}\ln P_{ij}$$

where *k* = 1/ln(*n*), *n* is the sample size and $P_{ij}=x_{ij}^{\prime }/\overset{n}{\underset{i=1}{\sum }}x_{ij}^{\prime }$.

Third, the URI and sub-resilience index $S_{i}$ of each city were calculated:

(4) $$S_{i}=\overset{m}{\underset{j=1}{\sum }}w_{j}\times x_{ij}^{\prime }$$

where $w_{j}=\frac{d_{j}}{\sum_{j=1}^{m}d_{j}}$, represents the weight of index *j*; $d_{j}=1-e_{j}$.

#### 2.2.2. Theil Index

Based on the concept of entropy in information theory, the Theil index was initially applied to the analysis of income gaps among individuals or regions; the total gap can be broken down into intragroup and intergroup gaps to more clearly identify its source [38]. In this paper, the unbalanced characteristics of the URI in the YRB were explored through the Theil index and its decomposition method, which is calculated as follows:

(5) $$T=T_{B}+T_{W}=\overset{K}{\underset{k=1}{\sum }}y_{k}\ln\left(\frac{y_{k}}{n_{k}/n}\right)+\overset{K}{\underset{k=1}{\sum }}y_{k}\left(\underset{i\in g_{k}}{\sum }\frac{y_{i}}{y_{k}}\ln\left(\frac{y_{i}/y_{k}}{1/n_{k}}\right)\right)$$

where *T*, $T_{B}$, and $T_{W}$ are the total gap, the intragroup gap, and the intergroup, respectively; *n* is the sample cities, which are divided into *K* groups (the authors of this paper divided the cities in the YRB into three groups: upper, middle, and lower reaches); each group is represented by $g_{k}$ (*K* = 1, 2, 3); $n_{k}$ is the number of cities in group *K*; $y_{k}$ is the ratio of the URI in group *K* to that in the YRB; and $y_{i}$ is the ratio of the URI of city *i* to the total.

#### 2.2.3. ESDA Model

##### Global Spatial Autocorrelation

The global autocorrelation analysis was used to verify whether there was a spatial aggregation of variables throughout the region by measuring the spatial interdependencies of observational data, characterized primarily by Moran’s *I* index. Thus, we used the global Moran’s *I* index to explore the spatial agglomeration of the URI in the YRB, and the formula [39] is as follows:

(6) $$I=\frac{n\sum_{i=1}^{n}\sum_{j=1}^{n}W_{ij}\left(x_{i}-\bar{x}\right)\left(x_{j}-\bar{x}\right)}{\sum_{i=1}^{n}\sum_{j=1}^{n}W_{ij}\sum_{i=1}^{n}{\left(x_{i}-\bar{x}\right)}^{2}}$$

where *n* is the number of research cities; *i* and *j* are two different research cities in the region; $x_{i}$ and $x_{j}$ are the average values of the URI of city *i* and *j*, respectively; and $\bar{x}$ is the average value of the URI of all cities. $W_{ij}$ is the spatial weight matrix: if *i* and *j* are adjacent, then $W_{ij}$ is 1, but if not, then $W_{ij}$ = 0. The range of *I* is [−1, 1], and when *I* > 0, it indicates positive spatial correlation; the larger the *I* value is, the stronger the spatial correlation and vice versa. The significance test of *I* was required, and the formula [40] is:

(7) $$Z\left(I\right)=\frac{1-E\left(I\right)}{\sqrt{Var\left(I\right)}}$$

where *Z*(*I*) is the significance level of Moran’s *I*, and *E*(*I*) and *Var*(*I*) are the mathematical expectation and variance, respectively. When *Z*(*I*) > 0 and is significant, it indicates positive spatial autocorrelation; when *Z*(*I*) < 0 and is significant, it indicates negative spatial autocorrelation.

##### Local Spatial Autocorrelation

To clarify the specific location of spatial agglomeration of high-value and low-value resilient cities within the YRB, we used the local Moran’s *I* index to identify the local spatial autocorrelation characteristics of urban resilience. The formula [41] is as follows:

(8) $$I_{i}=\frac{\sum_{i=1,j=1}^{n}W_{ij}\left(x_{i}-x\right)\left(x_{j}-x\right)}{S^{2}}$$

where $I_{i}$ is the local Moran’s *I* index of the *i*-th city and $S^{2}=\frac{1}{n}\overset{n}{\underset{i=1}{\sum }}{\left(x_{i}-\bar{x}\right)}^{2}$; the results also needed to be *Z*-tested, as in the formula above. At a certain level of significance, according to the significance level of the Moran’s *I* index and the results of the *Z* test, the research cities could be divided into four agglomeration relationships: (1) When Moran’s *I* was significant and positive and *Z*(*I*) > 0, it was a “High–High” (H–H) agglomeration relationship, and the URI of the research city and neighbouring cities is high; (2) when the Moran’s *I* index was significant and positive and *Z*(*I*) < 0, it was a “Low–Low” (L–L) agglomeration relationship, and the URI of the research city and neighbouring cities was low; (3) when the Moran’s *I* index was significant and negative and *Z*(*I*) > 0, it was a “High–Low” (H–L) agglomeration relationship, the URI of the research city was high, and the URI of the neighbouring unit was low; (4) when the Moran’s *I* index was significant and negative and *Z*(*I*) < 0, it was a “Low–High” (L–H) agglomeration relationship, the URI of the research city was low, and the URI of the neighbouring cities was high. When *Z*(*I*) = 0, it was randomly distributed.

#### 2.2.4. Geographical Detector Model

A geographical detector is a statistical model to distinguish spatial separation and reveal its influencing factors, including those of risk, ecological, and interaction [42]. In practical applications, it does not require too many assumptions, and influencing factor analysis offers advantages such as immune collinearity of multiple independent variables that have been applied in many fields in recent years [14,43]. The basic principle is to divide the total sample into several sub-samples and then use variance to judge spatial heterogeneity and variable relationships. If the sum of the sub-sample variances is less than the total variance of all samples, there is a spatial difference. If the spatial distribution of two variables tends to be consistent, there is a statistical correlation between the two variables. To consider the influence mechanism of urban resilience in the YRB, we constructed an index system that assessed urban resilience and identified the core influencing factors of URIs through factor detection and interaction detection in geographical detectors.

The main purpose of factor detection is to explore the degree of interpretation of the dependent variable *y* by the influence factor *x*, which is measured by the *q* value. The formula is:

(9) $$q=1-\frac{1}{N\sigma^{2}}\overset{L}{\underset{h=1}{\sum }}N_{h}\sigma_{h}^{2}$$

where *q* is the interpretive intensity of the influence factor on the URI with a range of [0, 1], and the larger the *q* value is, the stronger the spatial divergence of the dependent variable *Y*; if the spatial divergence is caused by the factor *X*, the larger the value of *q* is, so the interpretation of urban resilience is stronger; *h* is the stratification of factor *X* or variable *Y*; *N* is the sample size and $N_{h}$ is the number of cities in layer; and $\sigma^{2}$ and $\sigma_{h}^{2}$ are the variances of the whole area and *h* area, respectively.

Interaction detection is used to identify the interaction between different influencing factors $X_{i}$ and $X_{j}$. It can evaluate whether the factors $X_{i}$ and $X_{j}$ will increase or decrease the interpretation of the dependent variable *Y* when they work together, or whether the influence of these factors on the dependent variable Y is independent.

### 2.3. Data

**Figure 1:**
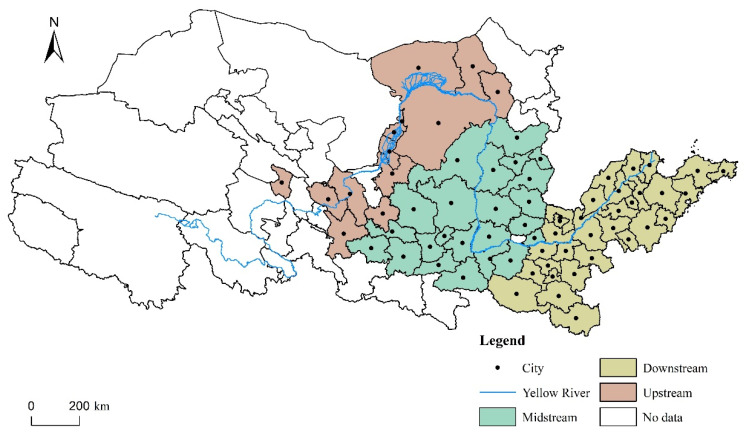
Sample area: the Yellow River Basin.

The research data could be divided into three main groups. (1) Socio-economic data: these data were mainly derived from the 2011–2018 “China City Statistical Yearbook”, “China City Construction Statistical Yearbook”, statistical yearbooks of provinces and cities, statistical bulletins of national economic and social development of cities, and individual missing data supplemented by interpolation or official local official websites. (2) Environmental data: annual precipitation and annual average temperature data were from the China Meteorological Data Network, and the original data were the monthly data from meteorological stations. After the abnormal values were removed, the annual average of the remaining stations was calculated, and the raster data of annual precipitation and annual average temperature were generated by kriging interpolation. MODIS NDVI data came from the Resource, Environment and Data Centre of the Chinese Academy of Sciences. (3) Geographical information data: based on the administrative cities mentioned in the YRB ecological protection and high-quality development strategy, the vector diagram of the administrative boundary of the YRB came from the National Basic Geographic Information Centre. Considering the integrity and continuity of the data, the authors of this paper excluded the Jiyuan, Zhongwei, Haidong, Linxia, Gannan Tibetan Autonomous, and Alxa regions. To better analyse the spatial heterogeneity and imbalance of urban resilience development in the YRB, the authors of this paper drew on existing research and divided it into three regions: the upstream, midstream, and downstream [44]. The study area is shown in Figure 1.

## 3. Spatiotemporal Evolution Characteristics of Urban Resilience

### 3.1. Temporal Evolution of URI

#### 3.1.1. Comprehensive Evolution Analysis

**Figure 2:**
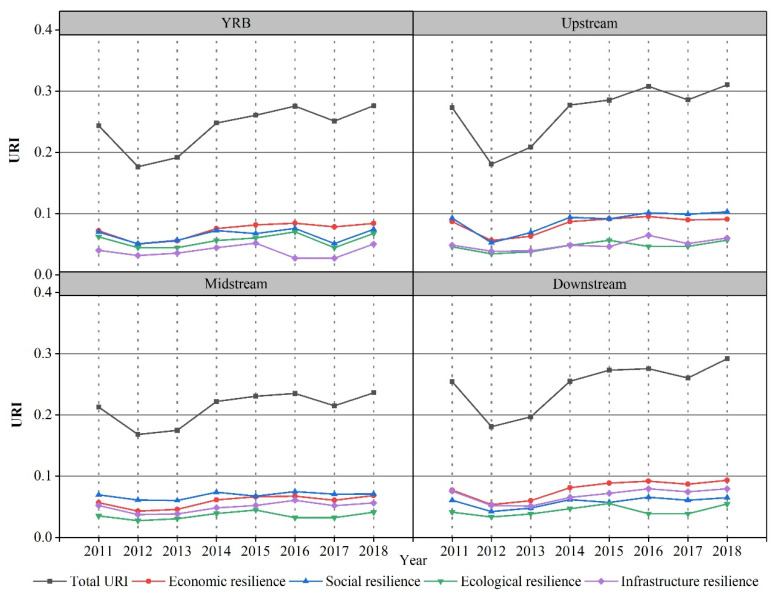
Temporal evaluation of the URI and subsystems in the YRB during 2011–2018.

Figure 2 shows that the URI of the YRB showed a “V”-shaped dynamic development trend from 2011 to 2018, with an increase of 13.4%. Its evolution can be characterized in two stages. In the first stage (2011–2012), the URI showed a downward trend from 0.2437 in 2011 to 0.1765 in 2012, a decrease of 27.6%. The reason for this decrease may be that in the post-financial crisis era, the development model oriented by resources and labour led to prominent contradictions in industrial structure, the low level of economic development led to less supply of social public goods, and pressure to “maintain growth” ignored the rational limited development of resources and environmental protection, thereby resulting in a serious ecological and environment problem and a significant decline in the URI. In the second stage (2012–2018), the URI showed a fluctuating upward trend from 0.1765 in 2012 to 0.2763 in 2018, an increase of 41.6%. During this period, the effectiveness of the “12th Five-Year Plan” gradually became apparent. The new round of development in the western region and the rise of the central region were implemented in depth. The construction of the main functional area began; it was proposed to optimize urbanization and significantly improve comprehensive urban carrying capacity, resilience, and risk resistance. Although the URI of the YRB has improved, the value remains relatively small, with obvious volatility and tortuousness. This phenomenon is directly associated with the weak economic strength of the cities in the YRB, their imperfect social systems and mechanisms, and the fragile ecological environment. These results indicate that there is a long road ahead to enhance urban resilience in the YRB.

The subsystems showed obvious volatility and hierarchical characteristics in the YRB from 2011 to 2018. The data distribution showed the characteristics of economic resilience > social resilience > ecological resilience > infrastructure resilience. At this stage, the URI of each subsystem improved, although the increases were quite different. Specifically, infrastructure resilience rose from 0.0399 to 0.0502, an increase of 26%; economic resilience rose from 0.0719 to 0.0841, an increase of 16.9%; ecological resilience rose from 0.0622 to 0.0677, an increase of 8.9%; social resilience rose from 0.0698 to 0.0743, an increase of 6.4%. The economic, social, ecological, and infrastructural resilience development of the YRB is obviously unbalanced, and it has also become an important factor restricting the improvement of comprehensive urban resilience. On the whole, the economic and social resilience index is high, while the infrastructure and ecological resilience index is low. The reason for such differences may be that the emergence of “urban diseases” has prompted the government to pay more attention to the adjustment and transformation of urbanization and industrialization, as well as issues related to social equity and stability such as education, medical care, and employment. However, the extensive resource-based cities in the YRB account for 47% of the total. They have not fundamentally eliminated the “resource curse” [45,46]. The negative environmental externalities caused by the transfer of high-polluting industries, coupled with these cities’ own poor ability to restore and purify ecological damage and environmental pollution, can easily transform these developed areas into “pollution refuges”.

In general, the coordinated and balanced development of various subsystems is a necessary measure to enhance urban resilience in the YRB. The malleable strategic framework of ecological protection and high-quality development will be able to strengthen the investment in and construction of infrastructure, as well as urban ecological and infrastructure resilience, under the guidance of ecological civilization.

#### 3.1.2. Regional URI Evolution

From 2011 to 2018, the comprehensive and subsystem URI of cities upstream, midstream, and downstream of the YRB increased (Figure 2). The comprehensive URI showed a “V”-shaped development trend, and the development of subsystem resilience significantly differed. The URI of upstream and midstream cities was found to be driven by “economy and society”, and the URI of downstream cities was found to be driven by “economy and infrastructure”. Specifically, in the upstream region, the comprehensive URI increased from 0.2731 in 2011 to 0.3104 in 2018, an increase of 13.7%, and the economic, social, ecological, and infrastructure resilience indices increased by 4.6%, 11%, 25%, and 24.4%, respectively. After 2013, the social resilience index surpassed the economic resilience index and became the dominant force in urban resilience. After 2016, the infrastructure resilience index surpassed the ecological resilience index. In the midstream region, the comprehensive URI rose from 0.2129 in 2011 to 0.2363 in 2018, an increase of 11%, and the URI of each subsystem had the most obvious hierarchy. The economic, social, ecological, and infrastructure resilience indices increased by 5.5%, 2.6%, 8.7%, and 12.4%, respectively. Unlike the other two regions, the ecological resilience index was lowest in the midstream. A probable reason for this is the midstream flow through the Loess Plateau, where soil erosion is the most serious. The ecological environment in this area is extremely sensitive and fragile, which, coupled with the high intensity of production, life, and resource development in many resource-based cities (as well as a disregard of the general natural laws of environmental protection and inefficient management) has resulted in the worst ecological resilience. In the downstream region, the comprehensive URI increased from 0.2543 in 2011 to 0.2917 in 2018, an increase of 14.7%, and the economic, social, ecological, and infrastructure resilience indices increased by 20.7%, 7.4%, 31.9%, and 4.9%, respectively. The economic resilience index was the highest and demonstrated a large increase. The high level of economic development drives urban infrastructure construction, so the infrastructure resilience index was the highest of the three regions. However, the coordination between economic growth and environmental protection is poor, and ecological resilience construction is insufficient.

#### 3.1.3. Difference Analysis Based on the Theil Index

In general, the Theil index dropped from 0.0877 to 0.0700 from 2011 to 2018 (Table 1), a decrease of 20.1%, indicating that the comprehensive URI gap is shrinking. Across time, in 2011–2012, 2013–2016, and 2017–2018, the difference in comprehensive URI narrowed, reaching a minimum of 0.0700 in 2018. In 2012–2013 and 2016–2017, the difference in comprehensive URI expanded, reaching the maximum of 0.0913 in 2013. The URI showed obvious volatility as a wave-like downward trend in the time series. 

In terms of the decomposition of regional differences, the Theil index in the upstream, midstream, and downstream areas declined during the study period, i.e., the regional differences in URI narrowed, with decreases of 34%, 21.2%, and 16.6%, respectively, and they showed a ladder-like rule of upstream > midstream > downstream. During this period, the mean contribution value and contribution rate of intraregional differences to the comprehensive URI difference were significantly greater than those of interregional differences. Compared with the beginning of the period, the intraregional differences decreased and the interregional differences slightly increased; thus, the differences in URI mainly reflected in intraregional differences. In summary, the comprehensive and intraregional differences in URI have diminished in fluctuations, while the changes in interregional differences are more complex. Therefore, in the construction of resilient cities, more attention should be given to regional coordination, linkage, and integrated development.

### 3.2. Spatial Pattern of URI

#### 3.2.1. Differentiation Characteristics of Comprehensive URI

**Figure 3:**
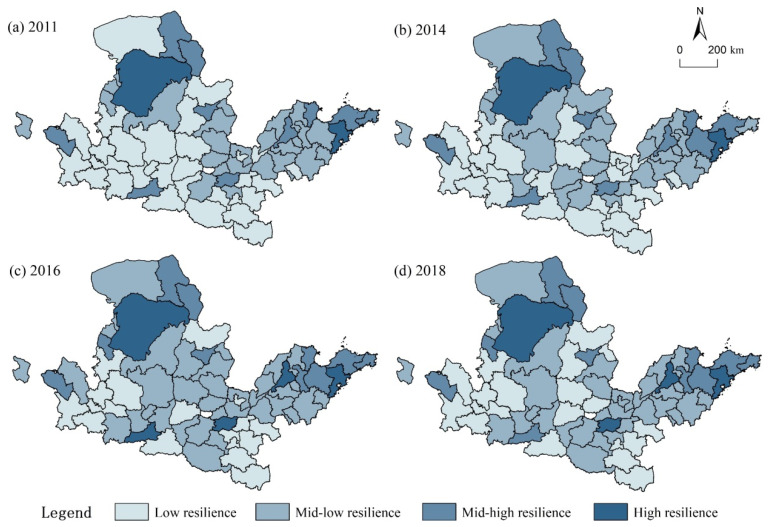
The spatial distribution of URI in the YRB from 2011 to 2018.

According to the results of the URI and referring to the classification standards of urban resilience in existing studies [35], the authors of this paper used 0.2, 0.35, and 0.5 as critical values to visualize the URI in 2011, 2014, 2016, and 2018 (Figure 3). *Y* ≤ 0.2 indicates low resilience, 0.2 < *Y* ≤ 0.35 indicates to mid-low resilience, 0.35 < *Y* ≤ 0.5 indicates mid-high resilience, and *Y* > 0.5 indicates high resilience.

**Figure 4:**
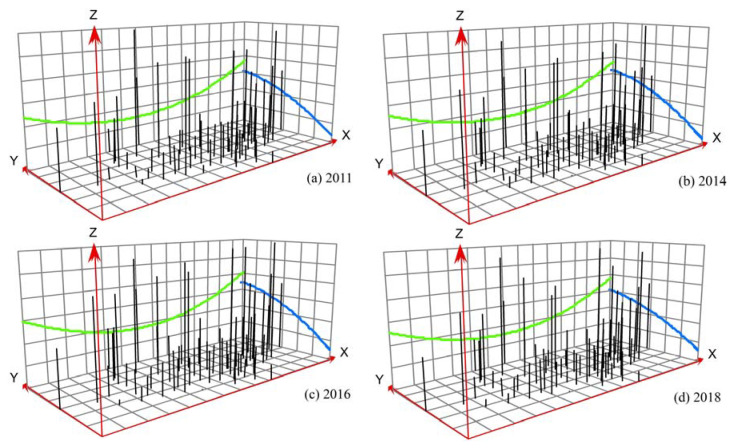
The evolution trend of URI in the YRB.

On the whole, the URI of the YRB has clustering characteristics, with regional central cities as the core and showing obvious zonal differences, as shown in Figure 4. First, the highly resilient cities are distributed, as indicated by dots in the figure. Before 2016, the cities of Ordos and Qingdao were representative, and Zhengzhou and Jinan also entered the ranks. The reason is that these cities have a significant “siphon effect” in the region. The collection of advantageous resources promotes the improvement of a city’s emergency mechanisms, and their considerable economic strength can bear the high cost of urban restoration. Moreover, social maturity and social vitality are high. Both increased labour costs and pressures on resources and the environment force cities to upgrade their industrial structure and technological transformation, which improves their environmental pollution prevention and control capabilities. Consequently, the ecosystem resilience increases, so the pressure URI is relatively high. Second, cities with mid-high resilience are mostly located on the fringes of cities with high resilience, such as Hohhot, Baotou, and Shizuishan in the upper reaches and Zibo, Weifang, and Yantai in the Shandong Peninsula; there are also a small number of cities distributed as indicated by dots in the figure, such as Lanzhou, Xi’an, and Taiyuan. Their resilience pattern was relatively stable during the study period. Finally, mid-low and low resilience cities accounted for 82.1% in 2011 and 77.6% from 2014 to 2018. They are widely distributed in the midwestern and southern regions and show certain spatial solidification characteristics. These are mainly small and medium-sized cities with poor basic conditions, insufficient economic growth momentum, and a low comprehensive URI.

#### 3.2.2. Spatial Correlation Analysis

##### Global Spatial Autocorrelation Analysis

The comprehensive URI of cities presents a positive spatial autocorrelation in the regional space; that is, cities with similar comprehensive resilience showed a spatial agglomeration trend. The Moran’s *I* index dropped from 0.211 in 2011 to 0.191 in 2018 (Table 2), indicating that the agglomeration trend weakened. From the perspective of subsystem resilience, economic resilience showed a positive spatial correlation, and both the Moran’s *I* value and the significance level are higher than other system resilience levels, again highlighting that economic resilience exerts the greatest impact on comprehensive URI while the impact of infrastructure resilience on comprehensive URI is second only to economic resilience. The low significance level and weak agglomeration trend of social and ecological resilience are additional evidence of the imbalanced social and ecological development among cities in the YRB.

##### Local Spatial Autocorrelation Analysis

The local autocorrelation test showed that in 2011, 2014, 2016, and 2018, 19.4%, 16.4%, 17.9%, and 16.4% of the cities, respectively, showed obvious positive spatial correlation (Table 3). Specifically, the urban resilience in “H–H” agglomeration areas was found to mainly be concentrated in the Shandong Peninsula Blue Yellow Economic Zone and the Hohhot–Baotou–Ordos urban agglomeration, including locations such as Weifang Yantai, Zibo, Hohhot, and Baotou. These cities have the advantages of good regional coordination and linkage. The radiation trickle effect of large cities such as Qingdao and Hohhot will drive economic growth and infrastructure construction. Together with the gradual improvement of the cross-regional coordinated governance pattern, the comprehensive ecological and environmental benefits have improved. The comprehensive resilience in the region is relatively high. Urban resilience in “L–L” agglomeration areas is distributed in the southern and southwestern parts of the YRB, such as Guyuan, Pingliang, Tianshui, Nanyang, Zhoukou, and Zhumadian. These cities have weak basic conditions and insufficient regional development endowments. Constrained by location, transportation, and resources, they have formed “depressions” for urban resilient development. Urban resilience in “L–H” and “H–L” agglomeration areas become fault areas in the radiation conduction of high and low-value areas. “H–L”-type cities such as Lanzhou and Xi’an have a strong siphon effect on the surrounding cities and are in the polarization stage of absorbing the collection of various resources around them, causing the resilience construction of the surrounding underdeveloped cities to comparatively lag. As a result, an “L–H” type of urban resilience development deficit has formed.

#### 3.2.3. Evolution Trend Analysis

To more intuitively illustrate the spatial evolution characteristics of URIs, ArcGIS 10.2 software was used to describe the spatial distribution trend of URIs in the YRB in 2011, 2014, 2016, and 2018 (Figure 4). In these visualisations, the *Z*-axis represents the URI, the line on the *X*-axis corresponds to the trend of the URI in the east–west direction, and that on the *Y*-axis indicates the north–south direction. On the whole, the curve of the URI from 2011 to 2018 has a relatively small range, and the overall stability remains steady. In the east–west direction, the curve presents an obvious U-shaped distribution pattern of high east–west and low central. This result indicates that cities in the east and west of the YRB have high resilience, whereas the central area has low urban resilience. In the north–south direction, the “high in the north and low in the south” curve trend is obvious, indicating that the URI of the northern part of the YRB is higher than that of the southern part, which shows significant regional differences.

### 3.3. Analysis of Influencing Factors of URI

#### 3.3.1. Index Selection

As shown by the analysis, there are significant spatiotemporal differences in URI throughout the YRB, and differences in the development of resilience among different cities and regions coexist with spatial correlation. As this area straddles the three major tectonic plates in China’s eastern, middle, and western regions, its socioeconomic development and natural background conditions are strongly imbalanced and the nonlinear interaction of various factors has led to a complicated spatiotemporal pattern of urban resilience. A review of the existing literature revealed that most of the current analysis on factors affecting urban resilience have focused on humanity and society and seldom involved the discussion of the impact of natural factors on urban resilience. The authors of this paper constructed an indicator system for multiple influencing factors of urban resilience in the YRB.

In terms of social and economic factors, the influencing effect of human factors was verified through the eight aspects of economic [47], infrastructure [8], fiscal [48], market [48], urbanization [48], financial [25], openness [48], and innovation factors [48]. According to the literature, we selected GDP/administrative area (*x*_1_), urban municipal public facilities construction/total investment in fixed assets (*x*_2_), fiscal revenue/GDP(*x*_3_), per capita retail sales of consumer goods (*x*_4_), urban population/total population (*x*_5_), financial institution deposit balance/loan balance (*x*_6_), total import and export/GDP (*x*_7_), and technology and education expenditure/GDP (*x*_8_) for characterization. Regarding natural factors, the impact of the natural environment on urban resilience was verified through the three aspects of water, air, and vegetation. The related variables were annual precipitation (*x*_9_), annual average temperature (*x*_10_), and vegetation index (*x*_11_). Jenks was used to discretize each explanatory variable into a type quantity. Based on factor and interactive detection in the geographical detector model, the main influencing factors of the URI in the YRB and the influence of the interaction of various factors on urban resilience were identified.

#### 3.3.2. Analysis of Influencing Factors

Through the use of factor detection to measure the interpretation strength of each influencing factor (*q*) (Table 4), the results showed that the influencing forces of each factor significantly varied, with the most influential factors including the economic (0.3891), fiscal (0.3181), market (0.748), urbanization (0.5875), openness (0.3471), and innovation factors (0.3803). Compared with social and economic factors, the significance level of natural factors was found to be low, as was their impact on URI.

Economic factors exert a significant impact on urban resilience. When citizens are attacked by man-made or natural disasters, robust economic strength can provide the necessary economic foundation and material guarantees for a city’s post-disaster recovery and development. In the early stages of urban development, excessive reliance on resource input reduced the spatial carrying capacity of the urban system, which weakened its ability to prevent external shocks and unknown risks and resulted in ecological environment system overloaded. Areas such as Changzhi, Xinzhou, Linfen, and Lvliang in Shanxi Province are important coal mine bases. Economic development mainly relies on resource-intensive industries, with low development quality and greater environmental protection pressure. In addition, in some cities such as Ordos, a large number of new district constructions have led to the oversupply of real estate development, imperfect infrastructure, and even “ghost cities”, causing great damage and pollution to the natural environment and seriously affecting urban resilience. With the transformation of the urban economic development model to high-quality intensification, technological progress and increased awareness of environmental protection have forced cities to improve their pollution control levels and the ability of urban systems to self-repair has increased. Such transformation has also provided capital accumulation for post-disaster recovery and strengthened cities’ resistance to various risk interferences. Therefore, efforts should be made to realize the linkage of the upstream, midstream, and downstream economies and industry synergy; promote the diversification of economic industries; further promote the transformation of economic development from quantitative expansion to qualitative improvement; and build a resilient economic structural system.

Fiscal factors play an important role in influencing urban resilience and exert an impact on the rise of resilience overall. The fiscal role is related to the government macro control. The larger the fiscal scale is, the more able the government is to centrally allocate social resources and provide strong human, material, and fiscal support for cities to resist external risks. The YRB mostly comprises small- and medium-sized cities in the middle of industrialization. The city scale is small, and most of the various elements are close to resource-based industries, which reduces the stability of the urban system and ecosystem in long-term development and leads to path dependence on the government’s fiscal support in the construction of urban resilience. However, if the fiscal scale exceeds the total amount of social surplus products, it will cause difficulty in capital turnover in the market, which is adverse for the optimal distribution of various resources in the urban system. Moreover, excessive government regulation is contrary to the general operational law of cities, easily produces rigidity in the process of urban development, and is not conducive to the construction of urban resilience. Therefore, in the future construction of resilient cities, the role of fiscal factors should be fully integrated to build an efficient service-oriented government and to improve the fiscal guarantee mechanism in the daily operation of the urban system.

Market factors exert a significant influence on urban resilience. Increased market capacity can strengthen the degree of regional marketization, stimulate the endogenous growth momentum of the urban economy, and enhance the resilience of local cities while promoting the resilience of surrounding areas through the radiation and driving effect of the market. In most underdeveloped small and medium-sized cities, residents tend to have a strong marginal willingness to consume, whereas their actual spending power is relatively weak. With the improvement of the urban social security level, residents’ burdens on medical care, education, and hygiene can be greatly reduced. This alleviation, in turn, can stimulate the conversion of residents’ income into production and living consumption, as well as provide a powerful impetus for urban economic development. In the process of building resilient cities, it is important to focus on improving the level of marketization and market activity, deepening the reform of the market-oriented allocation of factors, stimulating market potential, and improving the market’s emergency early warning mechanism to respond to disturbance threats. 

Urbanization factors exert a significant influence on urban resilience. An increase in the urbanization level means that the degree of urban agglomeration will increase. When a city is hit by disasters, it can quickly realize the allocation and complementation of various resources in the city, as well as reduce the degree of damage to the urban system. In recent years, the construction of new-type urbanization has intensified, the quality of urbanization has significantly improved, ecological cities and smart cities have improved the effectiveness of urban governance, and the operation of various functions in the urban system has been complemented and enhanced. The increase in urbanization has also strengthened the information communication within cities, avoiding the paralysis of the city system due to poor communication or “information failure”, improving the vitality of urban development, and strengthening the city’s ability to resist various shocks and threats. Therefore, urban planning and management should be coordinated to form a spatial pattern of urbanization with complete functions and the division of labour and coordination to provide good environmental support for improving urban resilience.

Openness factors exert a significant driving effect on urban resilience. According to the “pollution refuge hypothesis”, there are different environmental regulations among regions, and pollution-intensive industries show corresponding comparative advantages in different regions. When environmental regulations are relatively loose, the inflow of these polluting industries will increase the pressure on the local ecological environment, which is not conducive to the construction of ecological resilience. However, increasing the openness level is conducive to accumulating various resource elements and accelerating the diversified development of the industrial structure, with the effect of economies of scale thus becoming increasingly prominent. In the process of opening up, cities should acquire reverse knowledge spillovers, improve local technological innovation capabilities, and accelerate the optimization and upgrading of industrial structure to achieve balanced coordination between economic growth and environmental protection. In the construction of resilient cities, efforts should be made to build inland opening-up heights, actively guide the flow of funds to low-polluting and high-tech industries, and strengthen the introduction of talent and intellectual exchanges.

Innovation factors exert a significant influence on urban resilience. The achievements of scientific and technological innovation not only inject new momentum into economic development but also provide an effective means to solve ecological and environmental problems. From the perspective of science and technology, as localities pay increasing attention to science and technology investment, innovation dividends gradually appear. Technology spillovers are promoted through the network of city cooperation, which improves the efficiency of energy resource utilization and the input–output ratio, promotes the exploration and utilization of new energy, reduces the proportion of fossil energy consumption, improves the ability to control ecological environmental pollution, and reduces pollution emissions from industrial enterprises. In terms of education investment, the talent team is the key to technological innovation. Education investment helps to improve human capital, thereby improving the overall quality of workers, raising awareness of environmental protection, and providing a talent pool for the future development of the city, thereby strengthening the economic system and ecosystem anti-risk ability. All of these factors are highly beneficial to economic and ecological resilience.

From the perspective of other influencing factors: (1) The infrastructure factor (0.2101) showed a trend from significant to insignificant. Excessive government intervention is one possible reason [49]. Unreasonable planning and infrastructure investment have little effect on urban resilience, but the suboptimal allocation of the internal resource elements of a city system reduces the efficiency of resource use. However, this does not mean that infrastructure investment is negligible in the construction of urban resilience. Complete infrastructure is the basic material condition for cities to face various risks and challenges, and it provides basic support for the construction of resilient cities. In future infrastructure construction, more attention should be paid to improving the efficiency of infrastructure investment and building a multifaceted and systematic infrastructure pattern. (2) The financial factor (0.2404) was found to exert less impact on urban resilience than other factors and showed a downward trend, possibly because of the small financial scale of the cities in the YRB and the long-term dual economic structure. Idle funds in society are easily guided by policies to flow to highly resilient cities. Improving financial efficiency will intensify the competition of financial resources between high and low resilience cities, which will easily lead to financial risks. At present, the impact of the financial factor on urban resilience construction is limited.

Based on factor detection, the authors of this paper used interaction detection analysis to detect interaction types for impact factors (Table 5). The results showed that any two factors exerted a greater impact on urban resilience than a single factor, and the interaction types were all revealed to be nonlinear enhancements, indicating that urban resilience is affected by multiple factors. Specifically, the interaction between market factors and other influencing factors, such as infrastructure, fiscal, and urbanization, can better explain urban resilience. Compared to these, the impact of the other two factors is relatively low.

## 4. Conclusions

Based on the panel data of 67 cities in the YRB from 2011 to 2018, the temporal and spatial differentiation characteristics of urban resilience in the YRB were analysed, and a geographical detector model was used to analyse the influencing factors of urban resilience. The main conclusions are as follows:

(1) From 2011 to 2018, the comprehensive URI of the YRB was at a low-medium level, showed two-stage evolutionary characteristics, and presented a “V”-shaped dynamic fluctuation; it rose from 0.2437 to 0.2763, an increase of 13.4%. The resilience of each subsystem has obvious volatility and hierarchical characteristics, showing the following trend: economic resilience > social resilience > ecological resilience > infrastructure resilience.
(2) From the regional perspective, the comprehensive URI of the upstream, midstream, and downstream regions has somewhat increased, but the development and change of the resilience index of each subsystem in the region show significant regional characteristics. In addition, the comprehensive difference in URI in the basin has shown a shrinking trend. The intraregional differences in the three major sectors have shrunk, while the interregional differences have slightly increased. Intraregional differences are the main source of urban resilience differences.
(3) From the spatial analysis, we found that the URI of the YRB had obvious zonal differences from 2011 to 2018. The global Moran’s *I* index showed that the URI has presented a positive spatial autocorrelation in the regional space. In terms of local agglomeration, the “H–H”-type cities are mostly concentrated in the Shandong Peninsula and the Hohhot–Baotou–Ordos urban agglomeration, and the “L–L”-type cities are distributed in the southern and southwestern parts of the YRB. From the perspective of global trends, the U-shaped distribution trend in the east–west direction indicates that the urban resilience of the eastern and western parts of the YRB is significantly greater than that of the central region, and the upward trend from north to south indicates that the resilience of northern cities is greater than that in the south.
(4) From the analysis of influencing factors, we found that socioeconomic factors exert a greater influence on urban resilience than natural factors. Factor detection results showed that economic, fiscal, market, urbanization, openness, and innovation factors are the core impacts factors of urban resilience, while infrastructure and financial factors exert low impacts on urban resilience. In terms of interactive detection, the impact of the interaction factor on urban resilience was found to be significantly greater than that of a single factor, the interaction type of each interaction factor is characterized by nonlinear enhancement, and urban resilience in the basin is affected by multiple influencing factors.

## 5. Implications

The authors of this paper define urban resilience as a city’s ability to resist unknown risks and recover after a disaster. We used empirical analysis methods to explore the spatiotemporal evolution and influencing factors of urban resilience. This analysis provides a helpful reference for the prevention and resolution of major risks in the YRB against the background of the new era and provides new ideas for building resilient cities to promote sustainable development. In the process of urban development, the evolution of humanistic and natural elements or coupling with other elements constantly shapes the spatial form within the city, presenting multiscale characteristics within the spatiotemporal differentiation of urban resilience, which also makes the influencing factors of urban resilience more complex. At present, the urban resilience of the YRB is generally at a mid-low level, with large spatial and regional differences. The resilience of subsystems within the basin and among regions shows a clear hierarchical structure, reflecting a significant imbalance of economic, social, ecological, and infrastructure construction in the YRB. Achieving the coordinated development of the resilience of various subsystems has become the key to strengthening urban resilience. For this reason, cities in the YRB should strengthen the strategic top-level design of resilient city construction on the basis of their natural endowments and actual social and economic conditions; build effective economic development, social and public policy coordination, and infrastructure planning and construction mechanisms; strengthen the capacity of ecological and environmental protection; and establish a scientific and reasonable urban resilience system evaluation system. Considering that academia has not yet reached a consensus on the basic connotations of and evaluation criteria for urban resilience, future research will start with basic concepts to analyse the scientific connotation and framework structure of urban resilience and take a diversified and composite perspective on urban resilience development in various regions to find a reasonable and resilient city construction path.

## Figures and Tables

**Table 1:** Theil index measurement and contribution rate of URI in the YRB from 2011 to 2018.

Year	Comprehensive Difference	Intraregional Difference					Interregional Difference	
		Upstream	Midstream	Downstream	Contribution Value	Contribution Rate	Contribution Value	Contribution Rate
2011	0.0877	0.1131	0.0676	0.0791	0.0830	94.69%	0.0047	5.31%
2012	0.0842	0.0861	0.0985	0.0723	0.0836	99.30%	0.0006	0.70%
2013	0.0913	0.0983	0.1141	0.0683	0.0890	97.50%	0.0023	2.50%
2014	0.0788	0.0896	0.0614	0.0780	0.0754	95.63%	0.0034	4.37%
2015	0.0773	0.0911	0.0689	0.0688	0.0735	95.13%	0.0038	4.87%
2016	0.0735	0.0692	0.0601	0.0745	0.0690	93.83%	0.0045	6.17%
2017	0.0788	0.0890	0.0595	0.0738	0.0730	92.57%	0.0059	7.43%
2018	0.0700	0.0745	0.0532	0.0660	0.0641	91.55%	0.0059	8.45%
Mean	0.0802	0.0889	0.0729	0.0726	0.0763	95.02%	0.0039	4.98%

**Table 2:** The Moran’s *I* Index of URI in the YRB.

Year	Moran’s *I*				
	Comprehensive URI	Economic Resilience	Social Resilience	Ecological Resilience	Infrastructure Resilience
2011	0.211 ***	0.423 ***	0.005	0.047	0.210 ***
2012	0.248 ***	0.374 ***	0.069	−0.030	0.240 ***
2013	0.127 *	0.288 ***	−0.025	0.288 ***	0.188 ***
2014	0.221 ***	0.405 ***	0.026	0.118 *	0.167 **
2015	0.167 **	0.384 ***	0.011	0.152 **	0.173 **
2016	0.177 **	0.385 ***	0.015	0.032	0.093
2017	0.159 **	0.371 ***	0.009	0.032	0.129 *
2018	0.191 ***	0.365 ***	0.030	0.067	0.136 **

Note: ***, **, and * represent significance at the levels of 0.01, 0.05, and 0.1, respectively.

**Table 3:** Local spatial evolution of URI in the YRB.

Year	H–H	L–L	L–H	H–L
2011	Weifang, Yantai, Zibo, Binzhou, Hohhot, Baotou	Guyuan, Pingliang, Tianshui, Qingyang, Nanyang, Zhoukou, Zhumadian	Bayannaoer, Shizuishan, Xinzhou, Rizhao	Lanzhou
2014	Weifang, Yantai, Zibo, Hohhot, Baotou	Guyuan, Pingliang, Tianshui, Luohe, Nanyang, Zhumadian	Bayannaoer, Shizuishan, Xinzhou, Binzhou, Rizhao	Lanzhou, Xi’an
2016	Weifang, Yantai, Zibo, Baotou, Shizuishan	Guyuan, Pingliang, Tianshui, Luohe, Nanyang, Zhoukou, Zhumadian	Bayannaoer, Xinzhou, Binzhou, Laiwu, Rizhao	Lanzhou, Xi’an
2018	Weifang, Yantai, Zibo, Binzhou, Baotou	Guyuan, Pingliang, Tianshui, Nanyang, Zhoukou, Zhumadian	Bayannaoer, Shizuishan, Rizhao	Lanzhou

**Table 4:** Detection results of factors affecting urban resilience in the YRB.

Year	2011	2014	2016	2018	Average
*x*_1_	0.4089 ***	0.3382 **	0.4280 ***	0.3811 **	0.3891
*x*_2_	0.3139 *	0.2101	0.1133	0.2031	0.2101
*x*_3_	0.2102 **	0.2535 **	0.3798 ***	0.4288 ***	0.3181
*x*_4_	0.8401 ***	0.7454 ***	0.7889 ***	0.6167 ***	0.7478
*x*_5_	0.4189 ***	0.6586 ***	0.6513 ***	0.6212 ***	0.5875
*x*_6_	0.3042 ***	0.2230 ***	0.1888 **	0.2456 ***	0.2404
*x*_7_	0.2840 **	0.3252 *	0.4511 ***	0.3282 ***	0.3471
*x*_8_	0.4081 ***	0.4115 ***	0.2987 ***	0.4031 ***	0.3803
*x*_9_	0.0846	0.1578	0.0476	0.0579	0.0870
*x*_10_	0.1142	0.1194	0.1383	0.0766	0.1121
*x*_11_	0.1267 *	0.1335	0.0788	0.1376	0.1191

Note: ***, **, and * represent significance at the levels of 0.01, 0.05, and 0.1, respectively.

**Table 5:** Interaction of influencing factors on urban resilience in the YRB.

2011		2014		2016		2018	
Interaction Factor	Value	Interaction Factor	Value	Interaction Factor	Value	Interaction Factor	Value
*x*_3_ ∩ *x*_1_	0.6304	*x*_3_ ∩ *x*_1_	0.6274	*x*_3_ ∩ *x*_1_	0.6679	*x*_3_ ∩ *x*_2_	0.6490
*x*_4_ ∩ *x*_2_	0.9326	*x*_4_ ∩ *x*_3_	0.8677	*x*_4_ ∩ *x*_1_	0.8375	*x*_4_ ∩ *x*_1_	0.7714
*x*_4_ ∩ *x*_3_	0.9393	*x*_5_ ∩ *x*_1_	0.7629	*x*_4_ ∩ *x*_2_	0.8247	*x*_4_ ∩ *x*_3_	0.7264
*x*_5_ ∩ *x*_1_	0.6862	*x*_5_ ∩ *x*_2_	0.6895	*x*_4_ ∩ *x*_3_	0.9154	*x*_5_ ∩ *x*_1_	0.7160
*x*_5_ ∩ *x*_2_	0.6324	*x*_5_ ∩ *x*_3_	0.7912	*x*_5_ ∩ *x*_1_	0.7444	*x*_5_ ∩ *x*_2_	0.6429
*x*_6_ ∩ *x*_1_	0.6045	*x*_5_ ∩ *x*_4_	0.8273	*x*_5_ ∩ *x*_3_	0.7969	*x*_5_ ∩ *x*_4_	0.7069
*x*_6_ ∩ *x*_5_	0.6240	*x*_6_ ∩ *x*_4_	0.8022	*x*_5_ ∩ *x*_4_	0.8367	*x*_6_ ∩ *x*_4_	0.7295
*x*_8_ ∩ *x*_1_	0.6924	*x*_6_ ∩ *x*_5_	0.7378	*x*_7_ ∩ *x*_3_	0.6698	*x*_6_ ∩ *x*_5_	0.6770
*x*_8_ ∩ *x*_2_	0.6141	*x*_7_ ∩ *x*_3_	0.6112	*x*_8_ ∩ *x*_1_	0.6387	*x*_7_ ∩ *x*_3_	0.6085
*x*_8_ ∩ *x*_3_	0.6917	*x*_7_ ∩ *x*_5_	0.7491	*x*_8_ ∩ *x*_3_	0.7827	*x*_7_ ∩ *x*_4_	0.7099
*x*_8_ ∩ *x*_5_	0.6853	*x*_8_ ∩ *x*_4_	0.6445	*x*_8_ ∩ *x*_5_	0.6901	*x*_8_ ∩ *x*_3_	0.6054
*x*_8_ ∩ *x*_7_	0.6299	*x*_8_ ∩ *x*_5_	0.6945	*x*_8_ ∩ *x*_7_	0.6538	*x*_8_ ∩ *x*_5_	0.6578

Note: This table only lists the interaction factors with higher interaction values.

## Data Availability

The data presented in this study are openly available in National Bureau of Statistics, reference number 978-7-5037-9120-8, 978-7-5037-8770-6, 978-7-5037-8432-3, 978-7-5037-8082-0, 978-7-5037-7706-6, 978-7-5037-7350-1, 978-7-5037-7019-7, 978-7-5037-6754-8.

## Appendix A

**Table A1:** The evaluation index system of urban resilience in the YRB.

Target	Criterion Layer	Index Layer	Nature	Index Meaning	Reference
Urban resilience	Economic resilience	GDP per capita	+	Economic aggregate	Shi et al. [1,50]
		Per capita fiscal expenditure	+	Financial security	Shi et al. [1,50]
		The proportion of tertiary industry in GDP	+	Economic structure	Chen et al. [49]
		Per capita savings deposit balance	+	Financial capital	Chen et al. [49]
		Per capita investment in fixed assets	+	Investment intensity	Shi et al. [1,50]
		Number of industrial enterprises above designated size	+	Economic growth	Chen et al. [49]
	Social resilience	Number of college students per 10,000 people	+	Education level	Chen et al. [49]
		Number of hospital beds per 10,000 people	+	Medical investment	Kammouh et al. [22]
		Average salary of employees	+	Residence income	Shi et al. [1,50]
		Proportion of employment in tertiary industry	+	Employment structure	Shi et al. [1,50]
		Number of registered unemployed persons in urban areas	+	Unemployment structure	Shi et al. [1,50]
		Health technicians per 10,000 people	−	Health protection	Kammouh et al. [22]
	Ecological resilience	Park green area per capita	+	Environmental conservation level	Kammouh et al. [22]
		Green coverage rate in built-up area	+	Urban greening level	Kammouh et al. [22]
		Comprehensive utilization rate of industrial solid waste	+	Waste utilization	Chen et al. [49]
		Harmless treatment rate of urban domestic garbage	+	Environmental remediation	Chen et al. [49]
		Industrial wastewater discharge per unit GDP	−	Environmental pollution pressure	Chen et al. [49]
		Industrial smoke and dust emissions per unit of GDP	−	Waste emission intensity	Chen et al. [49]
	Infrastructure resilience	Road area per capita	+	Infrastructure construction	Chen et al. [49]
		Number of buses per 10,000 people	+	Level of transportation facilities	Kammouh et al. [22]
		Density of urban drainage pipes	+	Engineering support capability	Chen et al. [49]
		Number of Internet broadband users	+	Internet penetration	Kammouh et al. [22]
		Electricity consumption per capita	−	Electricity development level	Shi et al. [1,50]
		Number of mobile phone users at the end of the year	+	Communication sophistication	Kammouh et al. [22]
